# The Prevalence of Frascati-Criteria-Based HIV-Associated Neurocognitive Disorder (HAND) in HIV-Infected Adults: A Systematic Review and Meta-Analysis

**DOI:** 10.3389/fneur.2020.581346

**Published:** 2020-12-01

**Authors:** Jiaqi Wei, Jianhua Hou, Bin Su, Taiyi Jiang, Caiping Guo, Wen Wang, Yang Zhang, Biru Chang, Hao Wu, Tong Zhang

**Affiliations:** ^1^Center for Infectious Diseases, Beijing Youan Hospital, Capital Medical University, Beijing, China; ^2^Beijing Key Laboratory for HIV/AIDS Research, Beijing, China; ^3^Research Institute for International and Comparative Education, Shanghai Normal University, Shanghai, China; ^4^Department of Psychology, Shanghai Normal University, Shanghai, China

**Keywords:** HIV-associated neurocognitive disorder, Frascati criteria, prevalence, systematic review, meta-analysis

## Abstract

**Background:** The HIV associated mortality is decreasing in most countries due to the widespread use of antiretroviral therapy. However, HIV-associated neurocognitive disorder (HAND) remains a problematic issue that lowers the quality of life and increases the public health burden among people living with HIV. The prevalence of HAND varies across studies and selected samples. Therefore, we aimed to quantitatively summarize the pooled prevalence of Frascati-criteria-based HAND and to explore the potential demographic, clinical, and immunological factors.

**Methods:** A comprehensive literature search in PubMed/Medline, Web of Science, Embase, and PsycINFO was performed. A random-effects meta-analysis was conducted using the event rate (ER) for the estimation of the incidence of HAND. Subgroup meta-analyses were used to evaluate between-group differences in categorical variables. Meta-regression with the unrestricted maximum likelihood (ML) method was used to evaluate associations of continuous variables.

**Results:** Eighteen studies whose sample sizes ranged from 206 to 1555 were included in the final analyses. The estimated prevalence of HAND, ANI, MND and HAD were 44.9% (95% CI 37.4–52.7%), 26.2% (95% CI 20.7–32.7%), 8.5% (95% CI 5.6–12.7%), 2.1% (95% CI 1.2–3.7%), respectively. Factors associated with HAND were percent female, current CD4 count, education level and country development level (all *ps* < 0.05).

**Conclusion:** Longitudinal cohort and multimodal neuroimaging studies are needed to verify the clinical prognosis and the underlying neurocognitive mechanism of HAND. In addition, it is urgently necessary to establish a standardized HAND diagnostic process.

## Introduction

According to a report from the World Health Organization (WHO), the number of people living with HIV (PLWH) around the world rose to approximately 37.9 million at the end of 2019. With the widespread use and earlier initiation of ART among PLWH, life expectancy has been dramatically prolonged in those who are well-compliant with ART (1, 2). However, the neurological involvement in HIV disease progression remains problematic in well-controlled PLWH, resulting in worsened quality of life and increased public health burden.

HIV-associated neurocognitive disorder (HAND) is one of the most prevalent comorbidities in the era of ART. According to the Frascati criteria, HAND is roughly categorized into three stages: asymptomatic neurocognitive impairment (ANI), mild neurocognitive disorder (MND), and HIV-associated dementia (HAD) (3). PLWH were systematically evaluated by (1) cognitive tasks in verbal/language, attention/working memory, abstraction/executive, memory, processing speed, sensory-perceptual, and motor skills, (2) everyday functioning, (3) other pre-existing conditions that related to cognitive function (such as major depression and substance use) (4). However, these criteria did not specify what psychological instruments should be used in HAND assessment, which leads to large heterogeneity in the adopted battery (5, 6). The reported HAND prevalence ranges from 21% (7) to 86% (8). Factors that are associated with the prevalence of HAND range from demographic (e.g., age, sex distribution, education, and country) to clinical (e.g., current/nadir CD4 T cell-count, HCV coinfection, and types of ART) and psychosocial variables [e.g., depression, anxiety, and stigma; (7, 9–25)]. However, the results are still mixed across studies. Although one meta-analytic report tried to determine the HAND condition among perinatally infected children (26), a meta-analytical study is still needed to address this gap in other populations (such as adults).

Thus, we aimed to synthesize the prevalence of HAND in adult PLWH and to explore the risk factors associated with HAND. In addition, we also aimed to pool the prevalence of HAND stages, which could provide a comprehensive picture for clinicians and policy makers. Furthermore, we provide suggestions for future implementation of the Frascati criteria.

## Methods

The work is reported according to the Preferred Reporting Items for Systematic Reviews and Meta-Analysis (PRISMA) guidelines [Supplementary Table 1; (27)]. This study is registered in PROSPERO (CRD42020156006, https://www.crd.york.ac.uk/prospero/#recordDetails).

### Search Strategy

A comprehensive literature search in PubMed/Medline, Web of Science, Embase, and PsycINFO was performed. Search terms were intersections of HAND-related terms (HIV-associated neuro-cognitive disorder OR HAND OR cognitive impairment) and disease terms (HIV OR AIDS). The reference lists of the selected articles and related review articles were further screened. The search was limited to English-written journal articles. The search time was limited from 2006 to 2020 due to the implementation of the Frascati criteria (5).

### Selection Criteria

To be included in the meta-analysis, the study had to evaluate the prevalence of HAND, or the prevalence of HAND could be calculated from the research. Studies were excluded if they were (1) case reports; (2) review articles or theoretical articles; (3) non-peer-reviewed materials; (4) analyses of fewer than 200 participants, as increased selection bias and insufficient power to detect HIV-associated dementia may exist in small-sized studies; (5) children-oriented; (6) purely assessments of psychometric properties; or (7) not analyzed by the Frascati criteria. Two reviewers (JH and JW) initially selected search results based on titles and abstracts. The remaining articles were further determined by full-text assessment by JHH and JQW. Disagreements between reviewers about eligibility were resolved by discussion with TZ.

### Data Extraction and Code

Two researchers independently extracted and coded data using an Excel spreadsheet. Outcomes of interest were the prevalence of HAND, and the secondary outcomes were the prevalence of the different stages of HAND. Other information was also extracted from articles, including article author, year of publication, patient education level, sex distribution, study location, mean age of participants, sample size, HCV condition, current CD4 T-cell count, nadir CD4 T-cell count, and time since HIV diagnosis.

### Data Analysis

We adopted Comprehensive Meta-Analysis (CMA) Version 2.0 (Biostat, Englewood, New Jersey) to conduct a quantitative analysis. First, the combined event rate (ER) was calculated using the number of HANDs and the total sample size. A random-effects meta-analysis was conducted using the event rate (ER) for the estimation of the incidence of HAND. The Begg rank correlation test was adopted to assess publication bias across studies when more than three comparisons were in the analysis (28). The trim and filled method was used to adjust for potential publication bias. *I*^2^ and *Q*-tests were used to assess the proportion and statistical significance of heterogeneity (29).

### Quality Assessment of Individual Studies

Two researchers using the Agency of Healthcare Research and Quality (AHRQ) methodology checklist (http://www.ncbi.nlm.nih.Gov/books/NBK35156/) for cross-sectional studies independently assessed the individual studies. The 11 items were as follows: (1) information source, (2) study criteria, (3) study period, (4) sampling, (5) interview method, (6) instrument validation, (7) exclusion criteria, (8) the measurement of confounding effects, (9) the process of dealing with missing values, (10) response rate, and (11) the use of follow-up assessment. Studies scoring over 8 points were regarded as high-quality studies, 4–7 points were moderate quality studies, and 0–3 points were low quality studies.

### Subgroup Analysis and Meta-Regression

Our primary outcome was HAND. The predefined categorical moderators were median/mean education level (below college vs. college or above), current median/mean CD4 T-cell counts (below 500 vs. 500 or above), mean/median nadir CD4 T-cell counts (below 200 vs. 200 or above), country development levels (developed, developing, or underdeveloped) which was classified according to the Human Development Index in the Human Development Report issued by the United Nations Development Program, time since HIV diagnosis (shorter than 120 months vs. longer than or equal to 120 months) and study quality (below 8 vs. 8 or above). Subgroup meta-analyses were used to evaluate between-group differences. The predefined continuous moderators were percent female and HCV-coinfection proportion. Meta-regression with the unrestricted ML method was used to evaluate associations. We considered the results with ps < 0.05 as significance-level factors and those with 0.05 < *ps* < 0.1 as trend-level factors.

## Results

### Characteristics of the Included Studies

**Figure 1:**
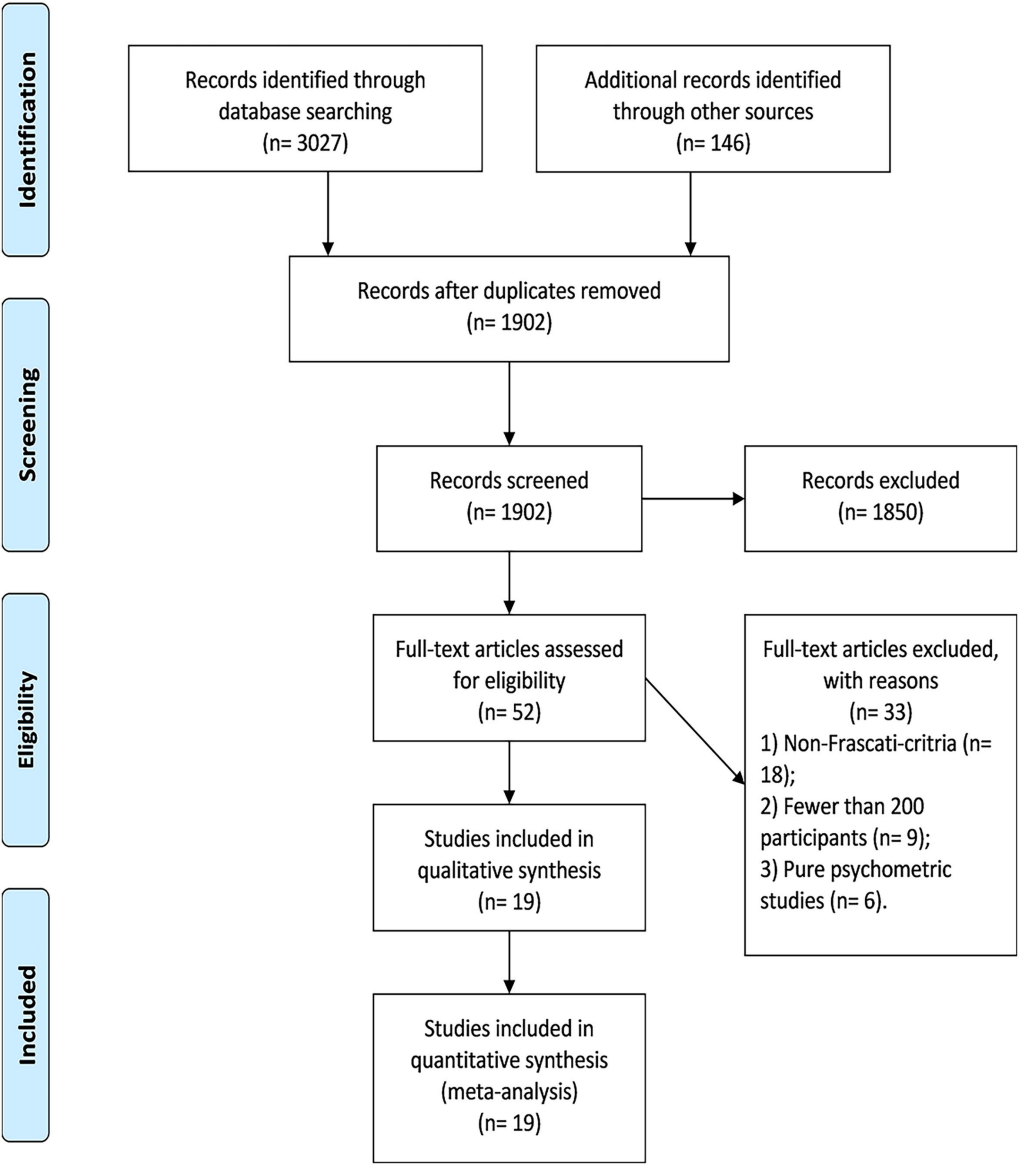
Selection of trials for inclusion of the systematic review and meta-analysis.

Overall, we identified 19 eligible studies in this review, whose sample size ranged from 206 to 1,555. Thirty-three studies in full-text assessment were excluded because they (1) did not use the Frascati criteria (*n* = 18), (2) had fewer than 200 participants (*n* = 9), and (3) were purely psychometric studies (*n* = 6) (Supplementary Table 2). The flowchart of study selection is shown in Figure 1.

Of the 19 studies, 13 (68.4%) studies were conducted in developed countries, 4 (21.1%) in developing countries and 2 (10.5%) in underdeveloped countries. Additionally, 4 studies (21.1%) were regarded as high-quality studies. Other characteristics of the included studies are shown in Table 1.

**Table 1:** Study characteristics.

**Study name**	**Country**	**Period of sampling**	**Age, year**	**Percent female**	**Education, year**	**Current/nadir CD4, cells/mm^**3**^**	**Hepatitis C Virus (HCV), *n***	**ART use proportion**	**Time since diagnosis, month**	**Study quality**
Mugendi-2019	Kenya	2015.7–2015.8	42	59	NA	446/NA	NA	NA	76	6
Wang-2019	China	1984–2004.9	39	NA	NA	506/NA	NA	NA	NA	5
Matchanova-2019	USA	NA	50	14	14	583/186	22	90%	190	4
Saylor-2019	Uganda	2013.8–2015.7	35	47	3	394/NA	NA	NA	NA	7
Santos-2019	Switzerland	2013.5–2016.11	53	20	13	638/180	NA	NA	NA	5
Gasco'n-2018	Brazil	2013.5–2015.2	45	32	12	626/NA	NA	NA	171	7
Haddow-2018	UK, Denmark, Belarus and Italy	2011.5–2013.1	46	16	NA	582/290	14	87%	119	7
Rodriguez-2018	Mexico	NA	61	14	13	NA/159	4	NA	133	5
Kallianpur-2018	USA	2003–2007	43	19	13	NA/177	24	73%	NA	8
Kinai-2017	Japan	2014.7–2016.7	46	5		550/163	4	97%	91	5
Yusuf-2017	Nigeria	NA	37	78	8	369/NA	NA	100%	54	4
Sacktor-2016	USA	2007–2012	47	NA	NA	589/330	13	NA	134	8
Bloch-2016	Australia	2011.10–2012.10	49	1	13	592/NA	4	92%	169	6
McDonnell-2014	England	2011–2012	45	0	16	550/NA	13	88%	59	6
Grant-2014	USA	NA	44	18	13	447/187	23	68%	NA	7
Bonnet-2013	France	2007.6–2009.11	47	21	12	515/260	6	89%	152	7
Ellis-2011	USA	NA	43	23	13	420/172	26	85%	120	6
Heaton-2010	USA	2003.9–2007.8	43	23	13	420/174	NA	85%	122	8
Metral-2019	German, French and Italian	2013.5–2016.11	55	20	13	634/180	17	NA	152	8

### HAND Prevalence

**Figure 2:**
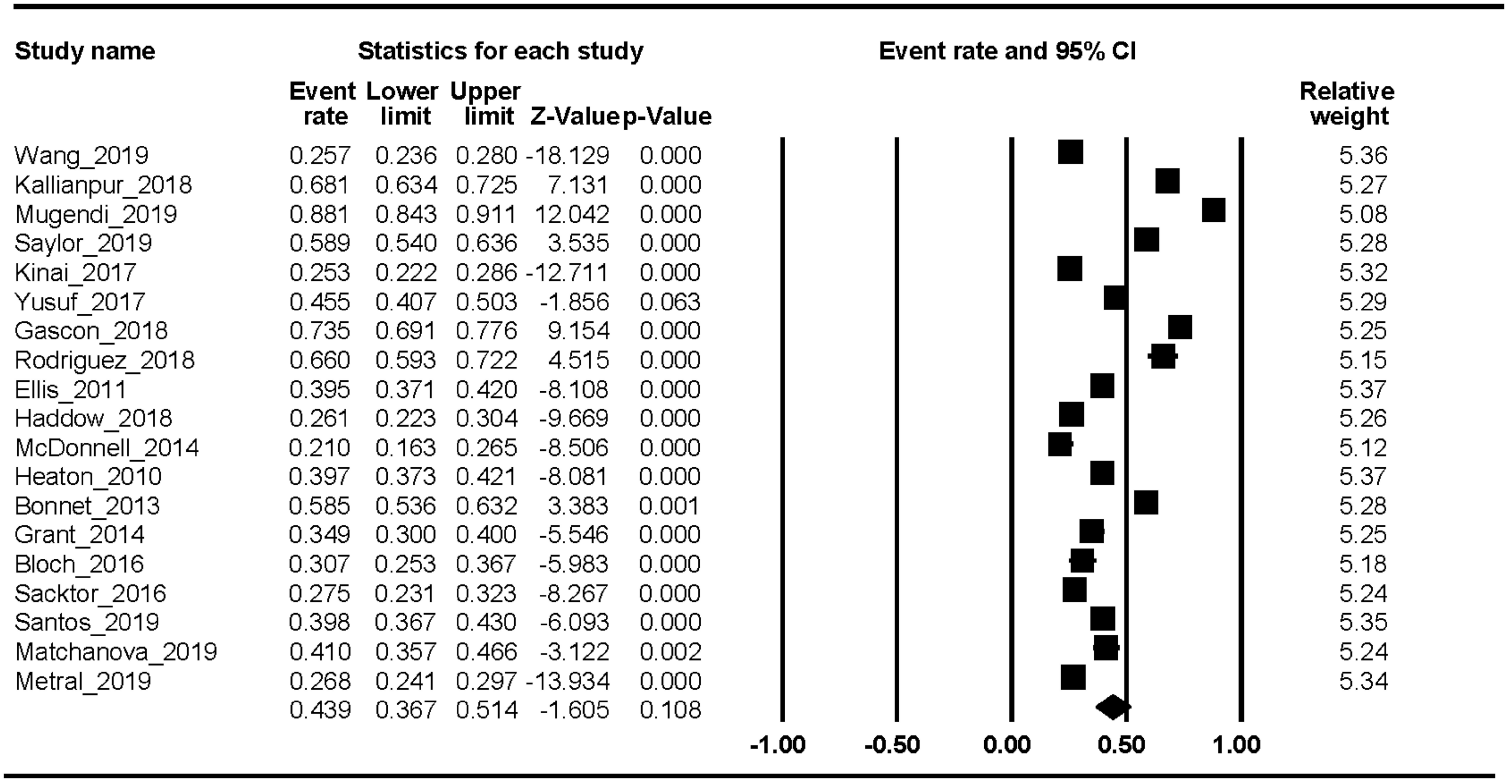
HAND.

Nineteen studies reported the HAND prevalence; therefore, the ER of each study could be analyzed. The Begg rank correlation test showed no significant publication bias (Kendall's tau = 0.216, *p* = 0.195). As shown in Figure 2, the combined ER of HAND was 43.9% (95% CI 36.7–51.4%). The results revealed significant heterogeneity across studies [*Q*_(18)_ = 1023.8, *p* < 0.001, *I*^2^ = 98.24%].

### Factors Associated With HAND

Factors associated with HAND with a significant level were percent female (beta = 0.021, *p* = 0.037, *n* = 17), current CD4 T-cell count [*Q*_(1)_ = 4.177, *p* = 0.041, *n* = 17], education level [*Q*_(1)_ = 43.15, *p* < 0.001, *n* = 15] and country development level [*Q*_(2)_ = 14.261, *p* < 0.001, *n* = 19]. Other factors not significantly associated with HAND were age, study quality, time since HIV infection, nadir CD4 count, HCV proportion, and ART use proportion (all *ps* > 0.05). Detailed information is shown in Table 2.

**Table 2:** Categorical variables associated with HAND.

**Independent variables**	**Point estimate^#^**	**95%CI**	***Q***	***P***
**Categorical variables**				
Education			43.153	<0.001
Below college	0.62	0.584–0.687		
College and above	0.315	0.258–0.379		
Nadir CD4			0.752	0.386
Lower than 200	0.419	0.312–0.534		
Higher than or equal to 200	0.365	0.241–0.51		
Current CD4			4.177	0.041
Lower than 500	0.526	0.4–0.648		
Higher than or equal to 500	0.352	0.275–0.438		
Study area			14.261	0.001
Under-developed	0.762	0.565–0.888		
Developing	0.525	0.37–0.675		
Developed	0.362	0.287–0.445		
Study quality			0.279	0.597
Low	0.45	0.363–0.54		
High	0.399	0.25–0.569		
**Continuous variables**				
Percent female	0.021	0.001–0.042	4.331	0.037
HCV proportion	0.041	−0.009 to 0.092	2.495	0.114
ART use proportion	−0.026	−0.057 to 0.017	1.118	0.29
Age	0.03	−0.045 to 0.108	0.643	0.422

# *Point estimate refers to event rate in categorical moderators and beta in continuous moderators*.

### HAD, MND, and ANI Prevalence

**Figure 3:**
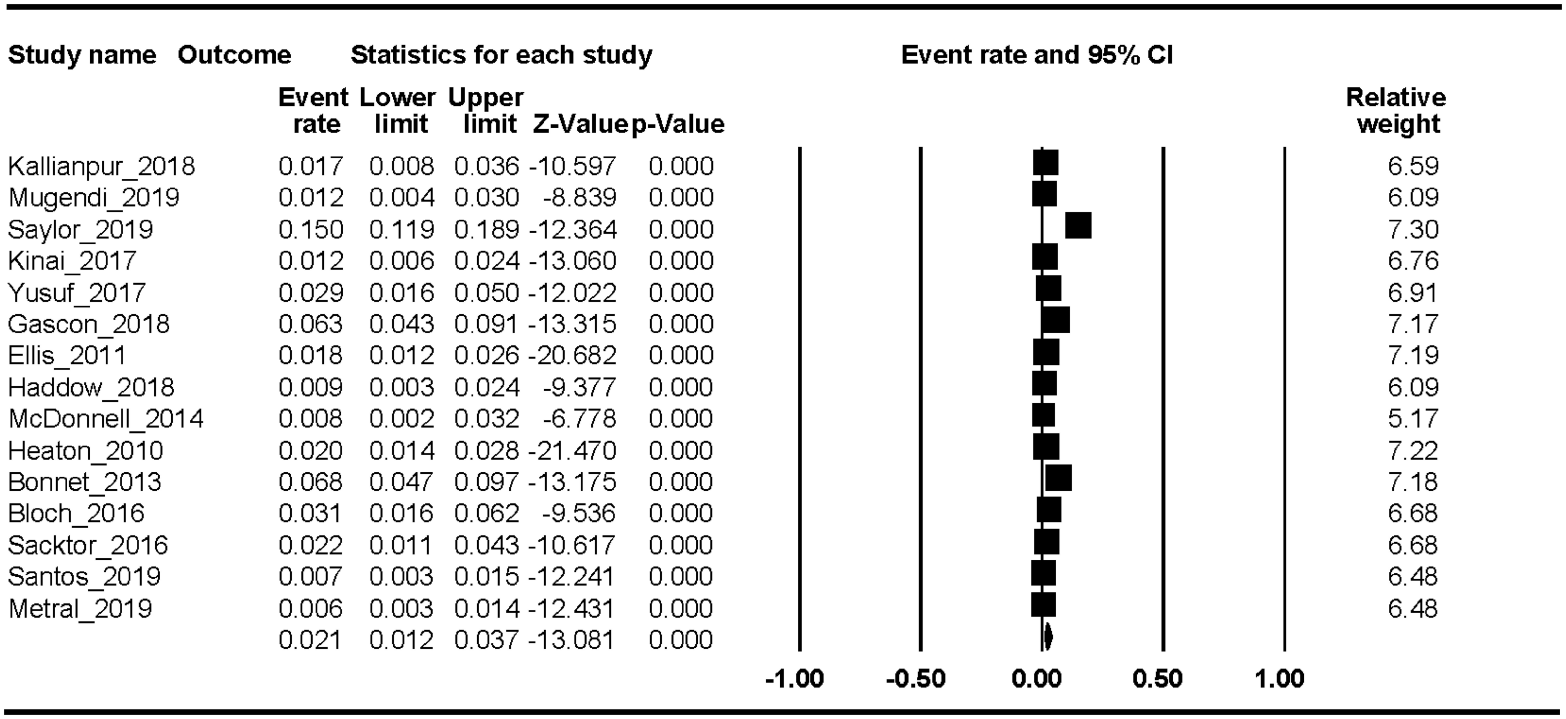
HAD.

Fifteen studies reported the HAD prevalence. The Begg rank correlation test showed no significant publication bias (Kendall's tau = −0.276, *p* = 0.151). As shown in Figure 3, the combined ER of HAD was 2.1% (95% CI 1.2–3.7%). The results revealed significant heterogeneity across studies [*Q*_(14)_ = 218, *p* < 0.001, *I*^2^ = 94.16%].

**Figure 4:**
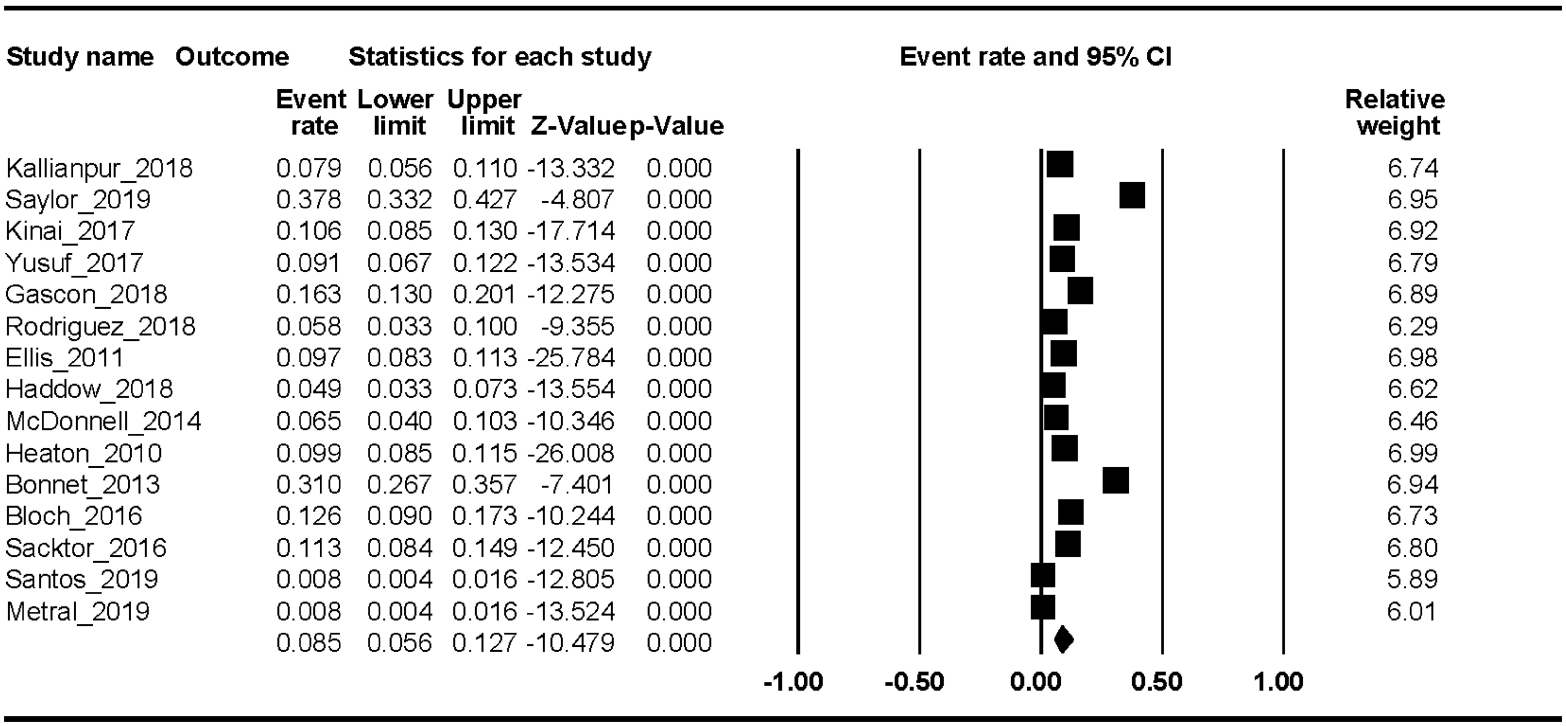
MND.

Fifteen studies reported the MND prevalence. The Begg rank correlation test showed no significant publication bias (Kendall's tau = −0.319, *p* = 0.112). As shown in Figure 4, the combined ER of MND was 8.5% (95% CI 5.6–12.7%). The results revealed significant heterogeneity across studies [*Q*_(14)_ = 508.37, *p* < 0.001, *I*^2^ = 97.25%].

**Figure 5:**
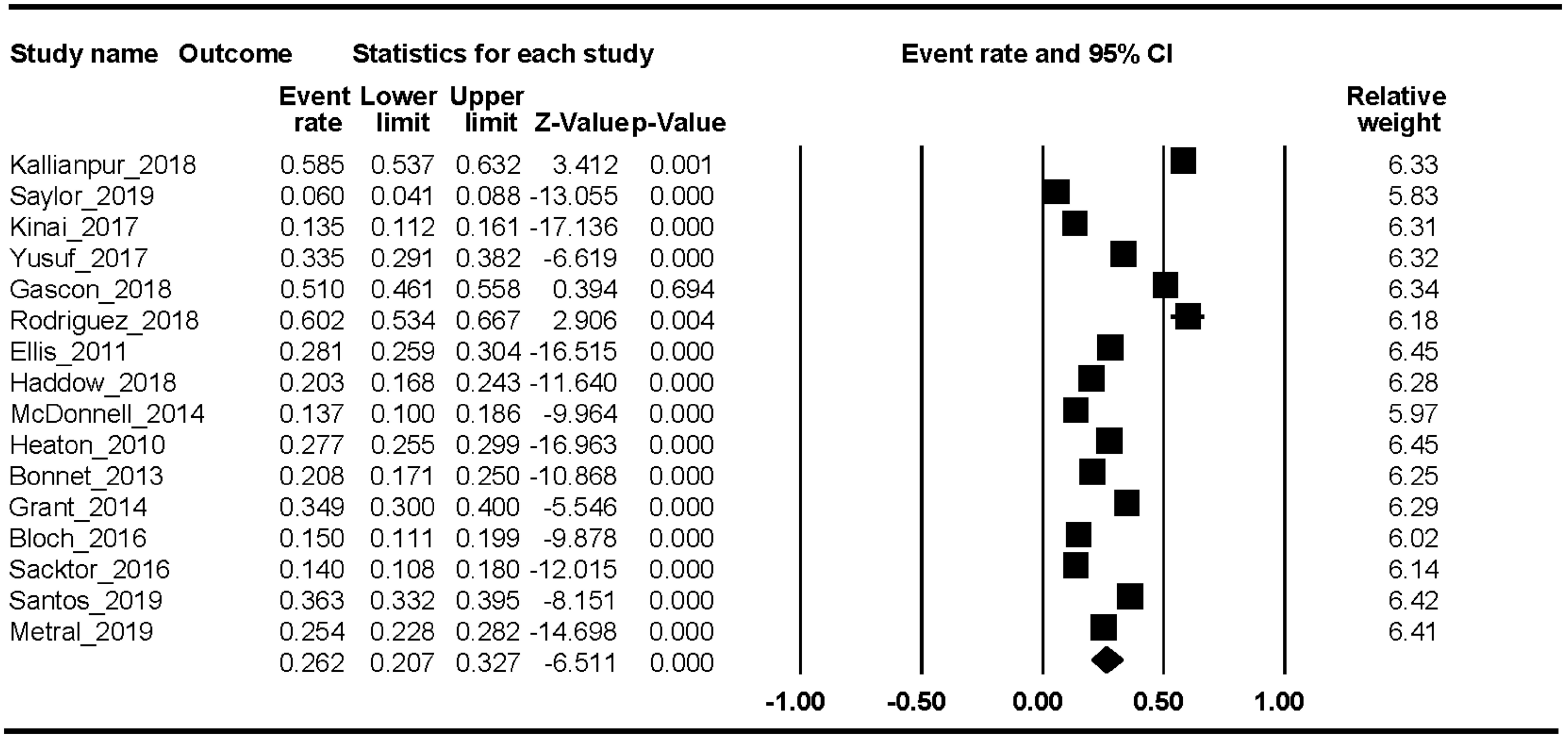
ANI.

Sixteen studies reported the ANI prevalence. The Begg rank correlation test showed no significant publication bias (Kendall's tau = −0.371, *p* = 0.054). As shown in Figure 5, the combined ER of ANI was 26.2% (95% CI 20.7–32.7%). The results revealed significant heterogeneity across studies [*Q*_(15)_ = 637.66, *p* < 0.001, *I*^2^ = 97.64%].

## Discussion

In this first meta-analysis of HAND, we found that the estimated prevalence was 43.9% (95% CI 36.7–51.4%). In addition, the pooled event rates of the different stages of HAND were ANI 26.2% (95% CI 20.7–32.7%), MND 8.5% (95% CI 5.6–12.7%), and HAD 2.1% (95% CI 1.2–3.7%). Factors associated with HAND were percent female, current CD4 T-cell count, education level, and country development level. The trend-level factors were nadir CD4 T-cell count and HCV proportion.

Regarding HAND prevalence, our systematic review found that nearly half of PLWH (43.9%) experienced Frascati-criteria-defined cognitive impairment, which is consistent with the results defined by other diagnostic criteria. This reflects that different diagnostic criteria may be comparable across settings. In addition, the prevalence of the different stages decreased with regard to the severity of HAND. However, a standardized assessment process with the same cultural-adaptive psychological instruments is still needed in clinical settings, and the current meta-analytical results (especially for ANI) should be carefully interpreted due to the seemingly large false-positive rate (30). In addition, almost no clinics or hospitals have integrated the screening and intervention of HAND into the usual care, which may lead to the missed diagnosis of HAND and worsening cognitive function status during HIV disease progression and ART.

With regard to the neurosubstrate for these results, several structural MRI studies have demonstrated that, compared to their general population counterparts, decreased gray matter volume (such as the prefrontal cortex and anterior cingulate cortex) (31–33) and white matter integrity (34, 35) in a large degree of brain networks exist in PLWH. Moreover, the ongoing HIV infection and inflammation of the CNS in the face of durable virus suppression may have a negative impact on the structure and function of the brain (36). Neurotoxicity of current therapeutic regimens (such as EFV-containing ART) may also negatively contribute to the dysfunction of brain networks (37). Thus, we could reasonably hypothesize that the biological and behavioral cascade caused by HIV infection may be a complex process accompanied by neurological, cognitive, and functional changes in the long run. In addition, future studies are needed to clarify the neuromechanism underlying HAND and the potential effects of other confounders (e.g., the type of treatment) related to brain function.

Our results have shown that factors significantly associated with HAND range from demographic to clinical variables. Education level is the central component of cognitive reserve that may have a protective impact on cognitive decline (38). A previous meta-analysis demonstrated that cognitive reserve was positively associated with cognitive performance among PLWH, with a large effect size (Cohen's *d* = 0.9) (39). Besides, we found that female was associated with higher odds of HAND, which is consistent with original reports in other populations (40–42). Sex difference may exist in prevalence, incidence, and cognitive decline trajectories and in certain type of cognitive impairment (such as non-amnesia MCI) (43). Surprisingly, no original studies reported the prevalence of HAND in transgender participants, which is highly under-representative in the minority group. However, hormone therapy, the commonly used treatment in transgender group, is associated with poor executive functioning (41, 44). Future studies are thus needed to verify the HAND prevalence and the effects of hormone therapy on cognition in transgender living with HIV. Higher current CD4 T-cell count is one of the representative biomarkers for immune reconstitution and a potential marker for lower viral titer in the CNS (45). Our results demonstrated that immune-function-restored PLWH may have decreased odds of HAND. Thus, this could be the therapeutic aim for PLWH with HAND symptoms in the future. In addition, country development level emerged as an influential factor in this meta-analysis. The underlying reasons for this association may be the poorer hygiene facilities, more neurotoxic treatment regimens, and lower awareness of HAND treatment in these underdeveloped countries and areas (46), which indicate an urgent need for the improvement of HAND care. However, we did not find a significant association between age and HAND prevalence. The narrow range of mean age (37–61) of included participants cannot represent all HIV participants especially the old-old and young participants. On the other hand, some of the included studies of high weights reported non-significant associations (11, 17, 25), which might also be the reason why the result is non-significant. Besides, we also failed to find the association between ART use condition and HAND prevalence. The possible reasonable might be the ceiling effects given that almost all studies reported a very high use of ART. However, it is hard to differentiate the effects of specific type of ART on HAND prevalence because most studies have reported different ART combinations.

Several limitations should be addressed in this meta-analysis: (1) a limited number of studies were included, which leads to small statistical power in subgroup analyses. However, the included studies adopted the same criteria (the Frascati criteria), which enhanced the method homogeneity and robustness of the meta-analytic results. (2) Studies adopted various instruments in each cognitive domain, which might lower the comparability across studies. Standardized cognitive assessment batteries for HAND diagnosis are needed in future studies.

## Conclusion

In summary, the prevalence of HAND is high, but the severest form of HAND (HAD) is rare among PLWH in the ART era. Demographic, clinical, and immunological factors are associated with the odds of HAND. Longitudinal cohort and neuroimaging studies are needed to verify the clinical prognosis and the underlying neurocognitive mechanism of HAND. In addition, it is urgently necessary to establish a standardized HAND diagnostic process.

## Data Availability Statement

All datasets presented in this study are included in the article/Supplementary Material.

## Author Contributions

JH and JW conceptualized the idea and drafted the manuscript. BS, JW, CG, BC, and TJ screened the articles and extracted data. JH, JW, WW, HW, and YZ conducted data analysis. TZ supervised the manuscript. All authors read and approved the final manuscript.

## Conflict of Interest

The authors declare that the research was conducted in the absence of any commercial or financial relationships that could be construed as a potential conflict of interest.
