# Supplementary material for: The Prevalence of Frascati-Criteria-Based HIV-Associated Neurocognitive Disorder (HAND) in HIV-Infected Adults: A Systematic Review and Meta-Analysis
Source: Front Neurol. 2020 Dec 1;11:581346. doi: 10.3389/fneur.2020.581346 (PMC7736554; doi:10.3389/fneur.2020.581346)
Supplement: Supplementary file 1 [file Table_2.DOC]

Page 1 of 2

| **Section/topic** | **#** | **Checklist item** | **Reported on page #** |
| --- | --- | --- | --- |
| Risk of bias across studies | 15 | Specify any assessment of risk of bias that may affect the cumulative evidence (e.g., publication bias, selective reporting within studies). | Page 5-6 |
| Additional analyses | 16 | Describe methods of additional analyses (e.g., sensitivity or subgroup analyses, meta-regression), if done, indicating which were pre-specified. | Page 6 |
| **RESULTS** | | |  |
| Study selection | 17 | Give numbers of studies screened, assessed for eligibility, and included in the review, with reasons for exclusions at each stage, ideally with a flow diagram. | Figure 1 |
| Study characteristics | 18 | For each study, present characteristics for which data were extracted (e.g., study size, PICOS, follow-up period) and provide the citations. | Page 7 and Table 1 |
| Risk of bias within studies | 19 | Present data on risk of bias of each study and, if available, any outcome level assessment (see item 12). | NA |
| Results of individual studies | 20 | For all outcomes considered (benefits or harms), present, for each study: (a) simple summary data for each intervention group (b) effect estimates and confidence intervals, ideally with a forest plot. | Page 7 and figure 2 |
| Synthesis of results | 21 | Present results of each meta-analysis done, including confidence intervals and measures of consistency. | Page 7 |
| Risk of bias across studies | 22 | Present results of any assessment of risk of bias across studies (see Item 15). | Page 7-8 |
| Additional analysis | 23 | Give results of additional analyses, if done (e.g., sensitivity or subgroup analyses, meta-regression [see Item 16]). | Page 7-8 and figure 2 |
| **DISCUSSION** | | |  |
| Summary of evidence | 24 | Summarize the main findings including the strength of evidence for each main outcome; consider their relevance to key groups (e.g., healthcare providers, users, and policy makers). | Page 8 |
| Limitations | 25 | Discuss limitations at study and outcome level (e.g., risk of bias), and at review-level (e.g., incomplete retrieval of identified research, reporting bias). | Page 10 |
| Conclusions | 26 | Provide a general interpretation of the results in the context of other evidence, and implications for future research. | Page 10 |
| **FUNDING** | | |  |
| Funding | 27 | Describe sources of funding for the systematic review and other support (e.g., supply of data); role of funders for the systematic review. | Page 11 |

*From:*  Moher D, Liberati A, Tetzlaff J, Altman DG, The PRISMA Group (2009). Preferred Reporting Items for Systematic Reviews and Meta-Analyses: The PRISMA Statement. PLoS Med 6(7): e1000097. doi:10.1371/journal.pmed1000097

For more information, visit: **www.prisma-statement.org**.

Page 2 of 2

**Table S2 Reasons for exclusion in full texts**

| 1 | Rukundo, G. Z. , & Namagga, J. K. . (2018). Prevalence and risk factors of hiv-associated neurocognitive disorders in rural southwestern uganda. *Journal of the Association of Nurses in AIDS Care,* *30*(5). | Not being diagnosed by Frascati criteria  Using IHDS screening instrument only cover 3 domains（motor speed, psychomotor speed and memory）and without testing everyday functioning and other pre-existing conditions. |
| --- | --- | --- |
| 2 | Megbaru, Debalkie, Animut, Muluken, Bekele, & Sorrie, et al. (2019). High prevalence of neurocognitive disorders observed among adult people living with hiv/aids in southern ethiopia: a cross-sectional study. *PloS one*. | Not being diagnosed by Frascati criteria  Using IHDS screening instrument only cover 3 domains（motor speed, psychomotor speed and memory）and without testing everyday functioning and other pre-existing conditions. |
| 3 | Belete, Tilahun, Medfu, Girmaw, & Yemiyamrew, Ephrem. . Prevalence of hiv associated neurocognitive deficit among hiv positive people in ethiopia: a cross sectional study at ayder referral hospital. *Ethiopian Journal of Health Sciences,* *27*(1). | Not being diagnosed by Frascati criteria  Using IHDS screening instrument only cover 3 domains（motor speed, psychomotor speed and memory）and without testing everyday functioning and other pre-existing conditions. |
| 4 | Tsegaw, M., Andargie, G., Alem, G., & Tareke, M. (2017). Screening HIV-associated neurocognitive disorders (HAND) among HIV positive patients attending antiretroviral therapy in South Wollo, Ethiopia. *Journal of psychiatric research*, *85*, 37-41. | Not being diagnosed by Frascati criteria  Using IHDS screening instrument only cover 3 domains（motor speed, psychomotor speed and memory）and without testing everyday functioning and other pre-existing conditions. |
| 5 | Kabuba, N. , Menon, J. A. , Franklin, D. R. , Heaton, R. K. , & Hestad, K. A. . (2016). Use of western neuropsychological test battery in detecting hiv-associated neurocognitive disorders (hand) in zambia. *AIDS and Behavior,* *21*(6), 1-11. | Not being diagnosed by Frascati criteria  Testing of validity or psychological instrument  Without testing other pre-existing conditions. |
| 6 | Mogambery, J. C. , Dawood, H. , Wilson, D. , & Moodley, A. . (2017). Hiv-associated neurocognitive disorder in a kwazulu-natal hiv clinic: a prospective study. *Southern African Journal of Hiv Medicine,* *18*(1). | Not being diagnosed by Frascati criteria  Without testing everyday functioning and other pre-existing conditions. |
| 7 | Joska, J. A. , Witten, J. , Thomas, K. G. , Robertson, C. , Casson-Crook, M. , & Roosa, H. , et al. (2016). A comparison of five brief screening tools for hiv-associated neurocognitive disorders in the usa and south africa. *AIDS and Behavior,* *20*(8), 1621-1631. | Not being diagnosed by Frascati criteria  Testing of validity or psychological instrument  Without testing everyday functioning and other pre-existing conditions. |
| 8 | Focà Emanuele, Paola, M. , Davide, M. , Silvia, C. , Salvatore, C. , & Andrea, B. , et al. (2016). Screening for neurocognitive impairment in hiv-infected individuals at first contact after hiv diagnosis: the experience of a large clinical center in northern italy. *International Journal of Molecular Sciences,* *17*(4), 434-. | Not being diagnosed by Frascati criteria  Without testing everyday functioning. |
| 9 | Barber, T. J., Bansi, L., Pozniak, A., Asboe, D., Nelson, M., Moyle, G., ... & Boffito, M. (2017). Low levels of neurocognitive impairment detected in screening HIV-infected men who have sex with men: the MSM Neurocog Study. *International journal of STD & AIDS*, *28*(7), 715-722. | Not being diagnosed by Frascati criteria  Without testing everyday functioning and other pre-existing conditions. |
| 10 | Zhao, T., Wei, B., Long, J., Tang, X., Zhou, M., & Dang, C. (2015). Cognitive disorders in HIV-infected and AIDS patients in Guangxi, China. *Journal of neurovirology*, *21*(1), 32-42. | Not being diagnosed by Frascati criteria  Without testing everyday functioning and other pre-existing conditions. |
| 11 | Troncoso, F. T., & Conterno, L. D. O. (2015). Prevalence of neurocognitive disorders and depression in a Brazilian HIV population. *Revista da Sociedade Brasileira de Medicina Tropical*, *48*(4), 390-398. | Not being diagnosed by Frascati criteria  Using IHDS screening instrument only cover 3 domains（motor speed, psychomotor speed and memory）. |
| 12 | McCombe, J. A., Vivithanaporn, P., Gill, M. J., & Power, C. (2013). Predictors of symptomatic HIV‐associated neurocognitive disorders in universal health care. *HIV medicine*, *14*(2), 99-107. | Not being diagnosed by Frascati criteria  Without testing everyday functioning and other pre-existing conditions. |
| 13 | Joska, J. A., Westgarth-Taylor, J., Myer, L., Hoare, J., Thomas, K. G., Combrinck, M., ... & Flisher, A. J. (2011). Characterization of HIV-associated neurocognitive disorders among individuals starting antiretroviral therapy in South Africa. *AIDS and Behavior*, *15*(6), 1197-1203. | Not being diagnosed by Frascati criteria  Without testing everyday functioning and other pre-existing conditions. |
| 14 | Cysique, L. A., & Brew, B. J. (2011). Prevalence of non-confounded HIV-associated neurocognitive impairment in the context of plasma HIV RNA suppression. *Journal of neurovirology*, *17*(2), 176-183. | Not being diagnosed by Frascati criteria  Without testing everyday functioning |
| 15 | Pumpradit, W., Ananworanich, J., Lolak, S., Shikuma, C., Paul, R., Siangphoe, U., ... & Valcour, V. (2010). Neurocognitive impairment and psychiatric comorbidity in well-controlled human immunodeficiency virus—infected Thais from the 2NN Cohort Study. *Journal of neurovirology*, *16*(1), 76-82. | Not being diagnosed by Frascati criteria  Enrollment is less than 200 |
| 16 | Vivithanaporn, P., Heo, G., Gamble, J., Krentz, H. B., Hoke, A., Gill, M. J., & Power, C. (2010). Neurologic disease burden in treated HIV/AIDS predicts survival: a population-based study. *Neurology*, *75*(13), 1150-1158. | Not being diagnosed by Frascati criteria  Without testing everyday functioning and other pre-existing conditions. |
| 17 | Simioni, S., Cavassini, M., Annoni, J. M., Rimbault, A. A., Bourquin, I., Calmy, A., ... & Du Pasquier, R. A. (2009). Prevalence of cognitive disorders in HIV plus patients with long-term suppression of viremia. In *9th International Symposium on NeuroVirology* (Vol. 15, pp. 91-91). | Not being diagnosed by Frascati criteria  Without testing everyday functioning and other pre-existing conditions. |
| 18 | Dufouil, C., Richert, L., Thiébaut, R., Bruyand, M., Amieva, H., Dauchy, F. A., ... & Dabis, F. (2015). Diabetes and cognitive decline in a French cohort of patients infected with HIV-1. *Neurology*, *85*(12), 1065-1073. | Insufficient data to calculate effect size |
| 19 | Sheppard, D. P., Iudicello, J. E., Bondi, M. W., Doyle, K. L., Morgan, E. E., Massman, P. J., ... & Woods, S. P. (2015). Elevated rates of mild cognitive impairment in HIV disease. *Journal of neurovirology*, *21*(5), 576-584. | Enrollment is less than 200 |
| 20 | Janssen, M. A., Meulenbroek, O., Steens, S. C., Góraj, B., Bosch, M., Koopmans, P. P., & Kessels, R. P. (2015). Cognitive functioning, wellbeing and brain correlates in HIV-1 infected patients on long-term combination antiretroviral therapy. *Aids*, *29*(16), 2139-2148. | Enrollment is less than 200 |
| 21 | Portilla, I., Reus, S., León, R., van-der Hofstadt, C., Sánchez, J., López, N., ... & Portilla, J. (2019). Neurocognitive Impairment in Well-Controlled HIV-Infected Patients: A Cross-Sectional Study. *AIDS research and human retroviruses*. | Enrollment is less than 200 |
| 22 | Awori, V., Nakigozi, G., Kisakye, A., Batte, J., Anok, A., Mayanja, R., ... & Sacktor, N. (2019). The Veterans Aging Cohort Study Index is not associated with HIV-associated neurocognitive disorders in Uganda. *Journal of neurovirology*, 1-5. | Enrollment is less than 200 |
| 23 | Fazeli, P. L., Casaletto, K. B., Paolillo, E., Moore, R. C., Moore, D. J., & HNRP Group. (2017). Screening for neurocognitive impairment in HIV-positive adults aged 50 years and older: Montreal Cognitive Assessment relates to self-reported and clinician-rated everyday functioning. *Journal of clinical and experimental neuropsychology*, *39*(9), 842-853. | Enrollment is less than 200 |
| 24 | Zhang, Y., Qiao, L., Ding, W., Wei, F., Zhao, Q., Wang, X., ... & Chen, D. (2012). An initial screening for HIV-associated neurocognitive disorders of HIV-1 infected patients in China. *Journal of neurovirology*, *18*(2), 120-126. | Enrollment is less than 200 |
| 25 | Kelly, C. M., van Oosterhout, J. J., Ngwalo, C., Stewart, R. C., Benjamin, L., Robertson, K. R., ... & Solomon, T. (2014). HIV associated neurocognitive disorders (HAND) in Malawian adults and effect on adherence to combination anti-retroviral therapy: a cross sectional study. *PloS one*, *9*(6), e98962. | Enrollment is less than 200 |
| 26 | Mazaheri, E. T. , Nejati, V. , Seyedalinaghi, S. , Dadras, O. , & Mohraz, M. . (2020). Prevalence of hiv-associated neurocognitive disorder (hand) and its subgroups among hiv-positive persons on anti-retroviral therapy in iran. Psihologija, 1-1. | Enrollment is less than 200 |
| 27 | Prevalence and factors associated with neuro cognitive disorders among HIV-positive patients in Ethiopia: A hospital-based cross-sectional study | Enrollment is less than 200 |
| 28 | Rosenthal, L. S., Skolasky, R. L., Moxley, R. T., Roosa, H. V., Selnes, O. A., Eschman, A., ... & Sacktor, N. (2013). A novel computerized functional assessment for human immunodeficiency virus-associated neurocognitive disorder. *Journal of neurovirology*, *19*(5), 432-441. | Testing of validity or psychological instrument |
| 29 | Mukherjee, T., Sakthivel, R., Fong, H. Y., McStea, M., Chong, M. L., Omar, S. F., ... & Cysique, L. A. (2018). Utility of using the montreal cognitive assessment (MoCA) as a screening tool for HIV-associated neurocognitive disorders (HAND) in multi-ethnic Malaysia. *AIDS and Behavior*, *22*(10), 3226-3233. | Testing of validity or psychological instrument |
| 30 | Milanini, B., Wendelken, L. A., Esmaeili-Firidouni, P., Chartier, M., Crouch, P. C., & Valcour, V. (2014). The Montreal Cognitive Assessment (MoCA) to screen for cognitive impairment in HIV over age 60. *Journal of acquired immune deficiency syndromes (1999)*, *67*(1), 67. | Testing of validity or psychological instrument |
| 31 | Gandhi, N. S., Moxley, R. T., Creighton, J., Roosa, H. V., Skolasky, R. L., Selnes, O. A., ... & Sacktor, N. (2010). Comparison of scales to evaluate the progression of HIV-associated neurocognitive disorder. *HIV therapy*, *4*(3), 371-379. | Testing of validity or psychological instrument |
| 32 | Tierney, S. M., Sheppard, D. P., Kordovski, V. M., Faytell, M. P., Avci, G., & Woods, S. P. (2017). A comparison of the sensitivity, stability, and reliability of three diagnostic schemes for HIV-associated neurocognitive disorders. *Journal of neurovirology*, *23*(3), 404-421. | Testing of validity or psychological instrument |
| 33 | Marin-Webb, V., Jessen, H., Kopp, U., Jessen, A. B., & Hahn, K. (2016). Validation of the international HIV dementia scale as a screening tool for HIV-associated neurocognitive disorders in a German-speaking HIV outpatient clinic. *PloS one*, *11*(12), e0168225. | Testing of validity or psychological instrument |
